# Protective Effects and Mechanisms of Polyethylene Glycol Loxenatide Against Hyperglycemia and Liver Injury in *db/db* diabetic Mice

**DOI:** 10.3389/fphar.2021.781856

**Published:** 2021-12-06

**Authors:** Yu Zhang, Yufeng Li, Junjun Zhao, Cong Wang, Bin Deng, Qilin Zhang, Chen Shi

**Affiliations:** ^1^ Department of Pharmacy, Union Hospital, Tongji Medical College, Huazhong University of Science and Technology, Wuhan, China; ^2^ Hubei Province Clinical Research Center for Precision Medicine for Critical Illness, Wuhan, China; ^3^ Preclinical Development Department, Shanghai Hansoh Biomedical Co., Ltd., Shanghai, China; ^4^ Pharmaceutical Research Institute, Jiangsu Hansoh Pharmaceutical Group Co. Ltd., Lianyungang, China

**Keywords:** GLP-1 receptor agonists, polyethylene glycol loxenatide, type 2 diabetes, liver injury, AMPK/ACC, PI3K/AKT

## Abstract

**Background:** Type 2 diabetes mellitus (T2DM) is a metabolic disorder with insulin resistance and impaired insulin secretion that can cause complications, including liver injury. Polyethylene glycol loxenatide (PEG-Loxe), a glucagon-like peptide-1 (GLP-1) analog, is widely used to treat T2DM. However, its specific glucose-lowering and hepatoprotective mechanisms of action have not been established yet.

**METHODS:** Using a high glucose-induced hepatocyte injury model and a type 2 diabetic *db/db* mouse model, we assessed PEG-Loxe’s impact on reducing blood glucose and improving liver injury in T2DM and revealed its mechanism.

**RESULTS:** PEG-Loxe treatment significantly reduced body weight and fasting glucose, increased glucose tolerance, improved serum and liver biochemical parameters (glycated hemoglobin, serum insulin, triglycerides, total cholesterol, high-density lipoprotein cholesterol, low-density lipoprotein cholesterol, alanine aminotransferase, and aspartate aminotransferase), and attenuated hepatic steatosis and liver and pancreatic tissue damages in *db/db* mice. Additionally, PEG-Loxe considerably inhibited oxidative stress, decreased pro-inflammatory factor (TNF-α, IL-6, and MCP-1) levels, and increased anti-inflammatory factor IL-10 levels. PEG-Loxe possibly inhibits hepatic lipid synthesis, oxidative stress, and inflammatory response by upregulating Sirt1, p-AMPK, and p-ACC expressions in the Sirt1/AMPK/ACC pathway of lipid metabolism, thereby improving T2DM liver injury. PEG-Loxe most likely also promotes GLP-1R expression by inhibiting β-cell apoptosis, which in turn activates the insulin PI3K/AKT pathway to promote insulin synthesis and secretion, thereby exerting hypoglycemic effects. *In vitro* cellular experiments further confirmed that PEG-Loxe possibly exerts hypoglycemic effects by activating the insulin PI3K/AKT pathway.

**Conclusion:** PEG-Loxe improved liver injury in T2DM probably by activating Sirt1/AMPK/ACC lipid metabolism pathway, and exerted hypoglycemic effects through activation of insulin PI3K/AKT pathway.

## Introduction

Type 2 diabetes mellitus (T2DM) is a metabolic disorder with insulin resistance (IR) and impaired insulin secretion. An estimated 642 million people in the world are projected to have diabetes by 2040 (Zheng et al., 2018). Long-term T2DM can lead to a variety of complications, including damage to the liver, kidney, cardiovascular system, and retina, and these are often huge economic and medical burdens on healthcare systems across the world (Regensteiner et al., 2015; Afkarian et al., 2016; Zheng et al., 2018). T2DM is currently treated primarily with oral hypoglycemic drugs and insulin. While traditional hypoglycemic drugs, including metformin, sulfonylureas, thiazolidinediones, α-glucosidase inhibitors, and insulin, may exert hypoglycemic effects through different mechanisms, they are prone to adverse effects, including hypoglycemia, weight gain, severe ketonuria, and lactic acidemia (Kohlroser et al., 2000; Monami et al., 2014; Fadini et al., 2017; Flory et al., 2020). Therefore, clinically, there is an urgent need for drugs with stable glucose-lowering effects and a low incidence of adverse effects.

Glucagon-like peptide-1 (GLP-1) is the most potent intestinal peptide hormone for insulin secretion that has been identified so far, and it is secreted primarily by L cells in the ileum and colon. It is now widely used to treat T2DM (Drucker et al., 2017). GLP-1 promotes intracellular insulin synthesis and secretion, inhibit glucagon secretion through binding to the GLP-1 receptor (GLP-1R) to facilitate the cellular signal transduction pathway (Madhu et al., 2020). Because GLP-1R is also expressed in the kidney, gastrointestinal tract, pancreas, nervous system, and heart, in addition to exerting hypoglycemic effects on the pancreas, GLP-1 can, hence, also suppress appetite, delay gastric emptying, increase insulin sensitivity in peripheral tissues and the liver, and protect the heart, brain, kidney, and liver (Drucker, 2018; Rowlands et al., 2018).

PEG-Loxe is a long-acting hypoglycemic agent derived from Exenatide via amino acid modification and PEGylation. PEG modification can reduce the toxicity, prolong the half-life and action time *in vivo*, thus improving bioavailability and the therapeutic effect. PEG-Loxe can effectively prolong GLP-1 activity and can be injected once a week due to its improved resistance to dipeptidyl peptidase-IV (DPP-IV) (Chen et al., 2017; Shuai et al., 2021). It is the first long-acting glucagon-like peptide-1 receptor agonist (GLP-1RA) in China, and can improve blood glucose in a glucose-concentration-dependent manner, making it less likely to trigger hypoglycemia. Results of a meta-analysis of 54 randomized controlled trials showed that PEG-Loxe reduced HbA1c in a similar way as exenatide, dulaglutide or liraglutide, and had an advantage in reducing the incidence of hypoglycemia (Jiang et al., 2021). PEG-Loxe was the only GLP-1RA to enhance the therapeutic dose without increasing the risk of hypoglycemia. Recently, GLP-1RA has been shown to effectively reduce lipid load and free fatty acid (FFA)-induced hepatic steatosis. However, their specific mechanisms of action for glucose-lowering and hepatoprotection are not well understood (Pan et al., 2021).

Islet dysfunction is an important factor in the development of hyperglycemia in patients with T2DM. Insufficient insulin secretion is caused by β-cell dysfunction, resulting in elevated blood glucose. Hyperglycemia, in turn, causes further damage to β-cells associated with a decrease in β-cell numbers due to apoptosis (Huang et al., 2007; Costes, 2018). Indeed, GLP-1 and its analogs can reduce β-cell endoplasmic reticulum (ER) stress and inhibit β-cell apoptosis (Ferdaoussi et al., 2008; Campbell and Drucker, 2013). The PI3K/AKT pathway is a key insulin signaling pathway that promotes glucose absorption and glycogen synthesis, thereby reducing blood glucose levels (Kuang et al., 2017; Wang et al., 2018). The Sirt1/AMPK/ACC axis, as demonstrated previously, is the master switch that controls the hepatic glucolipid metabolic pathway (Gruzman et al., 2009; Mottillo et al., 2016; Woods et al., 2017). However, the potential regulation of these signals by GLP-1 analogs or PEG-Loxe remains poorly understood.

All these data highlight the importance of PI3K/AKT, Sirt1/AMPK/ACC, and apoptosis in T2DM and the possible beneficial role of GLP-1 analogs in modulating these cellular response pathways to lower blood glucose and attenuate diabetes-related liver injury. In this study, we assessed PEG-Loxe’s pharmacological effects and its potential mechanisms of action on long-acting hypoglycemia, weight control, and improvement of liver complications by constructing animal and cellular models of diabetes mellitus. PEG-Loxe’s impact was also compared with the impact of short-acting GLP-1R agonists liraglutide and loxenatide to provide experimental evidence for the clinical application of PEG-Loxe.

## Materials and methods

### Chemicals and Reagents

Insulin receptor substrate-1 (IRS-1, #2382), p-IRS-1 (#2384), p-AKT (#4060), AKT (#9272), p-GSK-3β (#9322), GSK-3β (#12456), p-AMPK (#50081), AMPK (#5832), p-ACC (#11818), ACC (#3676), and Bax (#2772) were obtained from Cell Signaling Technology, Inc. (Danvers, MA, United States). PI3K (#ab133595), p-PI3K p85 (#ab182651), GLP-1R (#ab218532), Bcl-2 (#ab196495), carnitine palmitoyl transferase-1 (CPT1, #ab234111) and fatty acid translocase (FAT/CD36, #ab64014) were purchased from Abcam (Cambridge, MA, United States). GLUT4 (#AF5386), Caspase-3 (#AF6311), and Cleaved caspase-3 (#AF7022) were acquired from Affinity Biosciences (United States). Sirt1 (#13161-1-AP) was purchased from Proteintech Group (Wuhan, China). LY294002 (PI3K inhibitor, #1105) was obtained from Selleck Chemicals (United States). Horseradish peroxidase-conjugated goat anti-rabbit IgG, goat anti-mouse IgG, and β-actin were procured from Sigma-Aldrich (St Louis, MO, United States). Enzyme-linked immunosorbent assay (ELISA) kits for insulin were obtained from R&D Systems (Minneapolis, MN, United States). Biochemical analysis kits for glycosylated hemoglobin (HbA1c, H464-1), triacylglycerol (TG, A110-1), total cholesterol (TC, A111-1), high-density lipoprotein cholesterol (HDL-C, A112-1), low-density lipoprotein cholesterol (LDL-C, A113-1), alanine aminotransferase (ALT, C009-2), aspartate aminotransferase (AST, C010-2), reactive oxygen species (ROS, E004-1-1), glutathione (GSH, A006-2), malondialdehyde (MDA, A003-1), superoxide dismutase (SOD, A001-3), and catalase (CAT, A007-1) were bought from the Nanjing Jiancheng Bioengineering Institute (Nanjing, China). ELISA kits for interleukin-10 (IL-10, ELK1143), interleukin-6 (IL-6, ELK1157), tumor necrosis factor-α (TNF-α, ELK1395), and monocyte chemotactic protein-1 (MCP-1, ELK7694) were obtained from ELK Biotechnology (Wuhan, China).

### Experimental Animals

Male mice on C57BL/6.BKS.Cg-Dock7m +/+ Lepr^db/J(000697)^ (C57BL/6-*db/db*) and C57BL/6-*m/m* background (8 weeks) were purchased from the Changzhou Cavins Experimental Animal Co., LTD. The mice were bred in a 12-h dark-light cycle SPF room in standard cages (5 mice/cage) at a temperature of 22 ± 1°C. All animals had *ad libitum* access to water and standard chow. All animal experiments were approved by the Institutional Animal Care and Use Committee of Tongji Medical College, Huazhong University of Science and Technology. Animal care and experimental procedures were conducted under the Guidelines of the Institutional Animal Care and Use Committee of Tongji Medical College and the National Institutes of Health Guide for the Care and Use of Laboratory Animals.

After 1 week of adaptive feeding, 10 healthy *m/m* mice and 50 *db/db* mice were divided into six groups of 10 each. Group I (control, NC): healthy mice. Group II (T2DM): *db/db* mice. Mice in the NC and T2DM groups were subcutaneously injected with an equal volume of saline every 3 days. Group III (PEG-Loxe-L): *db/db* mice, subcutaneous injection of PEG-Loxe (0.3 mg/kg) every 3 days. Group IV (PEG-Loxe-H): *db/db* mice, subcutaneous injection of PEG-Loxe (1 mg/kg) every 3 days. Group V (Lira): *db/db* mice, subcutaneous injection of Lira (0.4 mg/kg) once a day. Group VI (Loxe): *db/db* mice, subcutaneous injection of Loxe (0.3 mg/kg) once a day. Treatment lasted four consecutive weeks for all groups.

### Determination of Body Weight, Fasting Blood Glucose, and Oral Glucose Tolerance Test

Mice body weight and fasting blood glucose (FBG) were measured once a week. The oral glucose tolerance test (OGTT) was performed at the end of the experiment. Briefly, all mice were subjected to fasting for 12 h and then given glucose (2 g/kg) through gavage (American Diabetes Association, 2021). Blood glucose samples were collected from the tail tip at 0, 30, 60, 90, 120, and 150 min after gavage, and their levels were measured with a glucometer (Bayer, Germany) according to the manufacturer’s instructions.

### Sample Collection and Serum Biochemical Parameter Detection

The FBG of all mice was tested after 4 weeks before the mice were anesthetized with pentobarbital. Blood samples were then collected from the eye orbits of mice, and the mice were euthanized. Liver and pancreatic tissues were subsequently harvested and weighed. Some of the liver and pancreatic tissues were fixed with 10% paraformaldehyde and stored at −80°C for further analysis. Blood specimens were immediately centrifuged (1,200 g, 4°C, 15 min) to obtain serum from which the levels of TC, TG, HDL-C, LDL-C, HbA1c, and insulin were quantified using a fully automatic biochemical analyzer (BS-420, Mindray, China).

### Biochemical Analysis of Liver

Liver tissues were homogenized in 9× (wt/vol) ice-cold phosphate-buffered saline and centrifuged at 3,500 rpm for 15 min to collect the supernatants. Lipid (TC and TG), AST, ALT, oxidative factors (ROS, MDA, SOD, GSH, and CAT), and inflammatory factors (IL-10, TNF-α, IL-6, and MCP-1) in liver tissues were measured using ELISA kits according to the manufacturer’s instructions.

### Detection of Reactive Oxygen Species in the Liver

Detection of reactive oxygen species in the liver was performed as described previously (Zhang et al., 2021). Briefly, 10 μm-thick frozen liver tissue sections were obtained using a freezing microtome (CM 1900, Leica, Germany), incubated with 5 mmol/L fluorescently-labeled DHE for 30 min at 37 °C in a light-proof environment (the DHE was diluted at 1:1,000), and then stained with 4′,6′-diamidino-2-phenylindole (DAPI, AS1075, Aspen Biological, Wuhan, China). Images were taken using a fluorescent microscope (MicroPublisher, MP3.3-RTV-CLR-10, Q-IMAGING, Canada) at ×200 magnification. The average fluorescence intensity of DHE was quantified using Image-Pro Plus 6.0 (IPP, Media Cybernetics, Rockville, MD, United States). Results were expressed by the ratio of the fluorescence intensity of DHE-positive area to the DAPI.

### Liver and Pancreas Histopathological Analysis

Liver and pancreas tissues were fixed in 10% formalin solution, dehydrated, and embedded with paraffin. Embedded liver and pancreas sections (3–5 μm thick) were stained with hematoxylin and eosin (H&E) for histopathological analysis (Wang et al., 2016). Besides, liver sections were stained with Sirius red and oil red O (ORO) (Wang et al., 2016). All sections were observed and imaged at ×400 magnification using an Olympus B×51 microscope (Tokyo, Japan).

### Immunohistochemistry

Paraffin-embedded pancreatic tissues (4 μm thick) were dewaxed, hydrated, and sealed with 5% bovine serum albumin (BSA) solution for 30 min at room temperature. The samples were then incubated with anti-insulin or anti-GLP-1R overnight and horseradish peroxidase (HRP)-conjugated anti-rabbit secondary antibody for 30 min the following day, stained with stable diaminobenzidine (DAB) solution, re-stained with hematoxylin, dehydrated, blocked, and observed and imaged using an Olympus BX51 microscope (Tokyo, Japan) (Zhou et al., 2020).

### Cell Culture and Treatment

Human hepatocellular carcinoma HepG2 cells were obtained from Tongji Medical College and cultured in DMEM medium containing 10% fetal bovine serum (FBS) and 1% penicillin-streptomycin in a 5% CO_2_ incubator at 37 °C. After growing and fusing to 70%, the HepG2 cells were randomly seeded in 6-well plates and grouped. Subsequently, they were exposed to 30 mM high glucose (HG) for 24 h, then to PEG-Loxe (100, 200 nM), Lira (100, 200 nM), or Loxe (100, 200 nM) with and without the addition of a PI3K inhibitor (LY294002, 20 μM), and treated for another 24 h. Cellular proteins were extracted to assay p-PI3K, PI3K, p-AKT, and AKT using Western blot.

### Western Blot Analysis

Western blot was performed as described previously (Zhou et al., 2020). Liver or pancreatic tissues were homogenized with RIPA lysate containing 1% PMSF protease inhibitor and phosphatase inhibitor, and total tissue protein was extracted via centrifugation for 10 min. Protein concentration was determined using the BCA kit. Equal amounts of proteins were separated with electrophoresis using 10–15% SDS-PAGE and then transferred to PVDF membranes where they were incubated with 5% BSA at room temperature for 3 h: liver tissues were incubated with primary antibodies p-IRS-1, IRS-1, p-PI3K, PI3K, p-AKT, AKT, p-GSK-3β, GSK-3β, GLUT4, p-AMPK, AMPK, p-ACC, ACC, Sir1, CPT1 and FAT/CD36 while pancreatic tissues were incubated with primary antibodies Bcl-2, Bax, Cleaved Caspase-3, and Caspase-3. After incubation, the membranes were washed three times with TBST solution, incubated with secondary antibodies at room temperature for 1 h, visualized with a chemically enhanced luminescence solution, and imaged using an automated imaging system (Gene Gnome5, Synoptics Ltd, United Kingdom). We assumed that β-actin was present at equal levels in all samples and served as a control.

### Statistical Analysis

All data were analyzed using GraphPad Prism version 7.0 (GraphPad Software, San Diego, CA, United States). Data are presented as the mean ± standard deviation, and their normal distribution was verified using the nonparametric Kolmogorov-Smirnov test. Differences between groups were analyzed using one-way ANOVA followed by Dunnett’s test. *p*-values < 0.05 were considered statistically significant.

## Results

### PEG-Loxe Reduces Body Weight and Hyperglycemia in T2DM Mice

**FIGURE 1:**
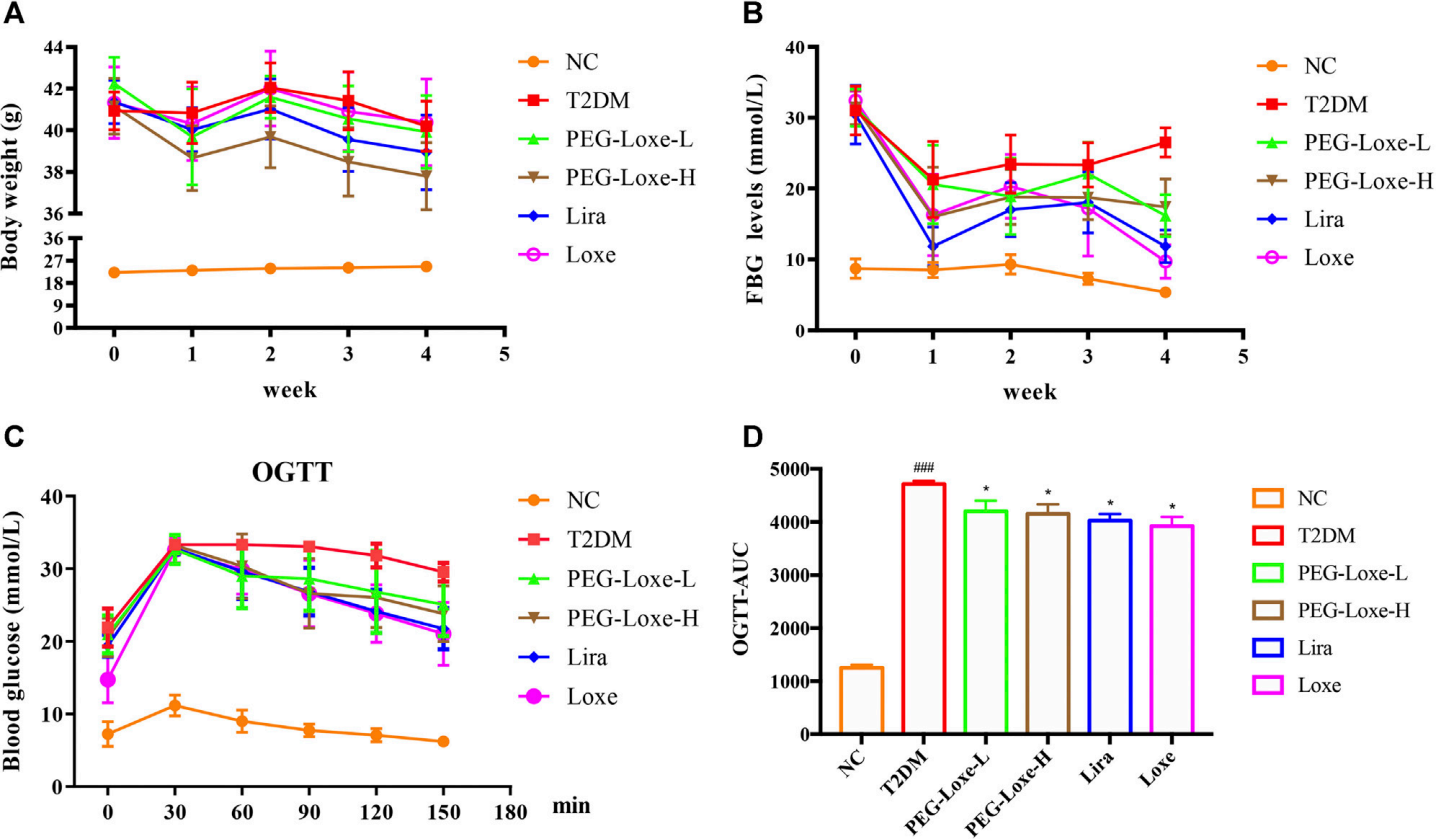
Polyethylene glycol loxenatide (PEG-Loxe) reduces body weight and hyperglycemia in *db/db* mice **(A)** Changes in body weight after PEG-Loxe treatment over 4 weeks **(B)** Changes in fasting blood glucose (FBG) levels after PEG-Loxe treatment over 4 weeks **(C)** Oral glucose tolerance test (OGTT) test **(D)** The area under the curve (AUC) of OGTT. Data are expressed as the mean ± SD (n = 8). ^###^ *p* < 0.001 compared to the normal control group; ^*^ *p* < 0.05 compared to the T2DM group.

As shown in Figure 1A, the body weights of mice in the T2DM group and Loxe groups did not change significantly before and after treatment. T2DM mice given PEG-Loxe-L, PEG-Loxe-H, and Lira had considerably reduced body weights. Of all the treatments, PEG-Loxe-H was most potent in reducing body weight.

FBG changes in mice were monitored weekly, and at the end of the experiment, FBG was markedly higher in T2DM group mice (26.51 mmol/L) than in NC group mice (5.39 mmol/L) (Figure 1B). However, treatment with PEG-Loxe-L, PEG-Loxe-H, Lira, and Loxe, the FBG of diabetic mice were significantly lower than that of T2DM group mice by 35.87, 36.51, 55.30, and 63.37%, respectively. Lira and Loxe showed more pronounced hypoglycemic effects than PEG-Loxe, probably because Lira and Loxe were administered daily while PEG-Loxe, as a long-acting formulation, was administered only once every 3 days. In addition, HbA1c was substantially increased in diabetic mice compared to healthy mice (*p* < 0.001). However, PEG-Loxe-H and Loxe reversed this increase (*p* < 0.01, Table 1).

**TABLE 1:** PEG-Loxe improved the levels of various biochemical parameters in *db/db* mice.

	NC	T2DM	PEG-Loxe-L	PEG-Loxe-H	Lira	Loxe
Serum TC (mmol/L)	1.08 ± 0.18	1.58 ± 0.26^###^	1.76 ± 0.21	1.50 ± 0.27	1.57 ± 0.26	1.37 ± 0.19
Serum TG (mmol/L)	0.62 ± 0.11	1.70 ± 0.33^###^	1.94 ± 0.34	1.26 ± 0.20^*^	1.31 ± 0.26^*^	0.98 ± 0.26^***^
Serum HDL-C (mmol/L)	1.38 ± 0.24	1.57 ± 0.09	1.84 ± 0.13^*^	1.84 ± 0.24^*^	1.77 ± 0.18	1.56 ± 0.10
Serum LDL-C (mmol/L)	0.35 ± 0.83	0.47 ± 0.14	0.51 ± 0.17	0.29 ± 0.08^*^	0.35 ± 0.06	0.44 ± 0.10
HbA1c (%)	4.44 ± 0.45	11.16 ± 1.12^###^	12.89 ± 1.62^**^	9.19 ± 0.93^**^	11.30 ± 0.80	9.03 ± 0.84^***^
Serum insulin (mIU/L)	6.52 ± 0.43	12.91 ± 0.40^###^	14.58 ± 1.70^*^	16.37 ± 0.90^***^	14.49 ± 0.63^*^	11.41 ± 2.02
Liver TC (mmol/gprot)	0.59 ± 0.26	6.55 ± 1.19^###^	4.18 ± 0.51^***^	1.40 ± 0.21^***^	3.02 ± 0.54^***^	3.76 ± 0.38^***^
Liver TG (mmol/gprot)	0.32 ± 0.19	2.74 ± 0.13^###^	1.84 ± 0.21^***^	0.81 ± 0.09^***^	1.35 ± 0.13^***^	1.88 ± 0.34^***^
Liver ALT (U/gprot)	3.48 ± 1.31	32.62 ± 3.35^###^	20.81 ± 3.46^***^	9.33 ± 0.72^***^	16.08 ± 2.18^***^	21.21 ± 1.86^***^
Liver AST (U/gprot)	9.75 ± 2.52	37.19 ± 3.84^###^	27.86 ± 2.23^***^	16.16 ± 1.89^***^	22.18 ± 3.23^***^	28.93 ± 3.68^***^

NC, denotes normal control group; T2DM, denotes type 2 diabetes group. Data are expressed as the mean ± SD (n = 8).

### *p* < 0.001 compared to the normal control group.

* *p* < 0.05.

** *p* < 0.01, and.

*** *p* < 0.001 compared to the T2DM, group.

OGTT was performed at the end of the experiment and revealed that diabetic mice given 2 g/kg of glucose via gavage had significantly higher plasma glucose levels at 30, 60, 90, 120, and 150 min (Figure 1C) compared to NC group mice, indicating a decrease in oral glucose tolerance in diabetic mice. In contrast, PEG-Loxe, Lira, and Loxe prevented the increase in blood glucose levels in diabetic mice, pointing to an improvement in impaired glucose tolerance (Figure 1C). The OGTT curve reflects the changes in glucose. The area under the curve (AUC) in T2DM mice was significantly greater than that in the NC group, but it was considerably reduced after PEG-Loxe, Lira, and Loxe treatments (Figure 1D).

### PEG-Loxe Improves Lipid Disorders in T2DM Mice

The effects of PEG-Loxe on serum and liver lipids were shown in Table 1. Compared to normal mice, T2DM mice had significantly higher (*p* < 0.001) levels of TC and TG in their serum and considerably higher levels of lipids in their livers (TC and TG). However, the changes in hepatic TC, TG and serum TG were markedly reversed after 4 weeks of treatment with PEG-Loxe-L, PEG-Loxe-H, Lira, and Loxe (*p* < 0.05). PEG-Loxe treatment, particularly the 1 mg/kg dose, also drastically elevated serum HDL-C levels and decreased LDL-C levels in T2DM mice.

### PEG-Loxe Improves Hepatic Steatosis and Liver Injury in T2DM Mice

**FIGURE 2:**
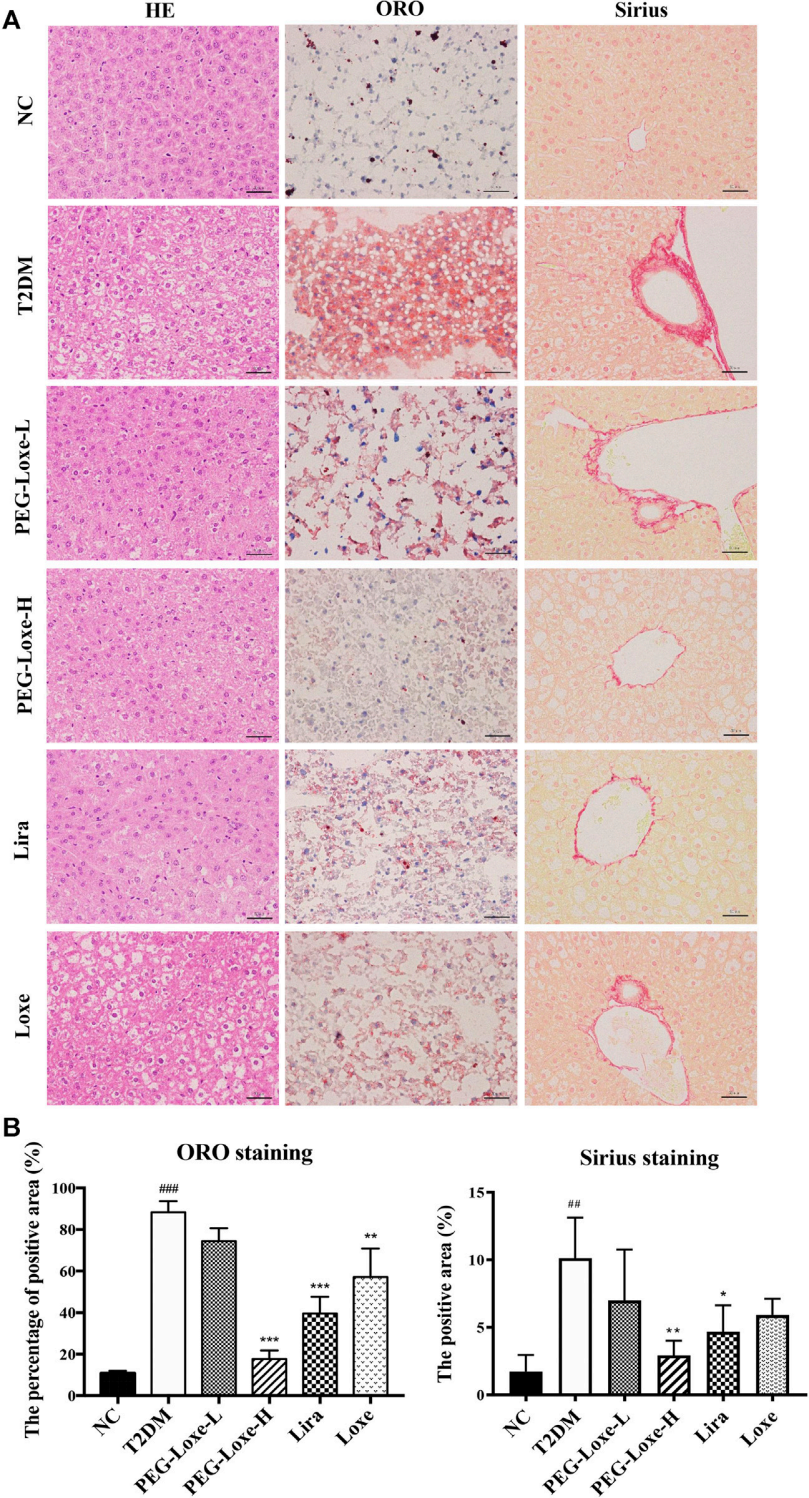
Effects of polyethylene glycol loxenatide (PEG-Loxe) on the histopathological changes in the liver of *db/db* mice **(A)** Representative images of the hematoxylin and eosin (H&E), oil red O (ORO), and Sirius staining of liver tissues (400×) **(B)** Quantitative analysis of liver injury using ORO and Sirius staining. Data are presented as the mean ± SD; n = 3; ^##^ *p* < 0.01 and ^###^ *p* < 0.001 compared to the normal control group; ^*^ *p* < 0.05, ^**^ *p* < 0.01, and ^***^ *p* < 0.001 compared to the T2DM group.

The H&E staining of liver sections of mice in the NC group revealed regular morphology, uniform distribution, and tight hepatocyte arrangements. T2DM mice exhibited cytoplasmic vacuolation and hepatocyte necrosis. However, PEG-Loxe-L, PEG-Loxe-H, Lira, and Loxe significantly reduced liver lesions after 4 weeks of treatment (Figure 2A). Measurements of the levels of hepatic ALT and AST to evaluate liver damage in T2DM mice established that T2DM mice had severe liver damage compared to normal controls, as evidenced by the significantly increased levels of ALT and AST (*p* < 0.001). However, PEG-Loxe, Lira, and Loxe treatments considerably alleviated these abnormal levels (Table 1).

In addition, Sirius red- and ORO-stained liver sections showed large lipid droplet depositions and liver fibrosis in the hepatocytes of T2DM mice, which were substantially reduced after 4 weeks of treatment with PEG-Loxe, Lira, and Loxe (Figures 2A,B). These results suggested that PEG-Loxe, Lira, and Loxe were protective against T2DM-induced liver injury.

### PEG-Loxe Attenuates Hepatic Oxidative Stress and Inflammatory Response in T2DM Mice

**FIGURE 3:**
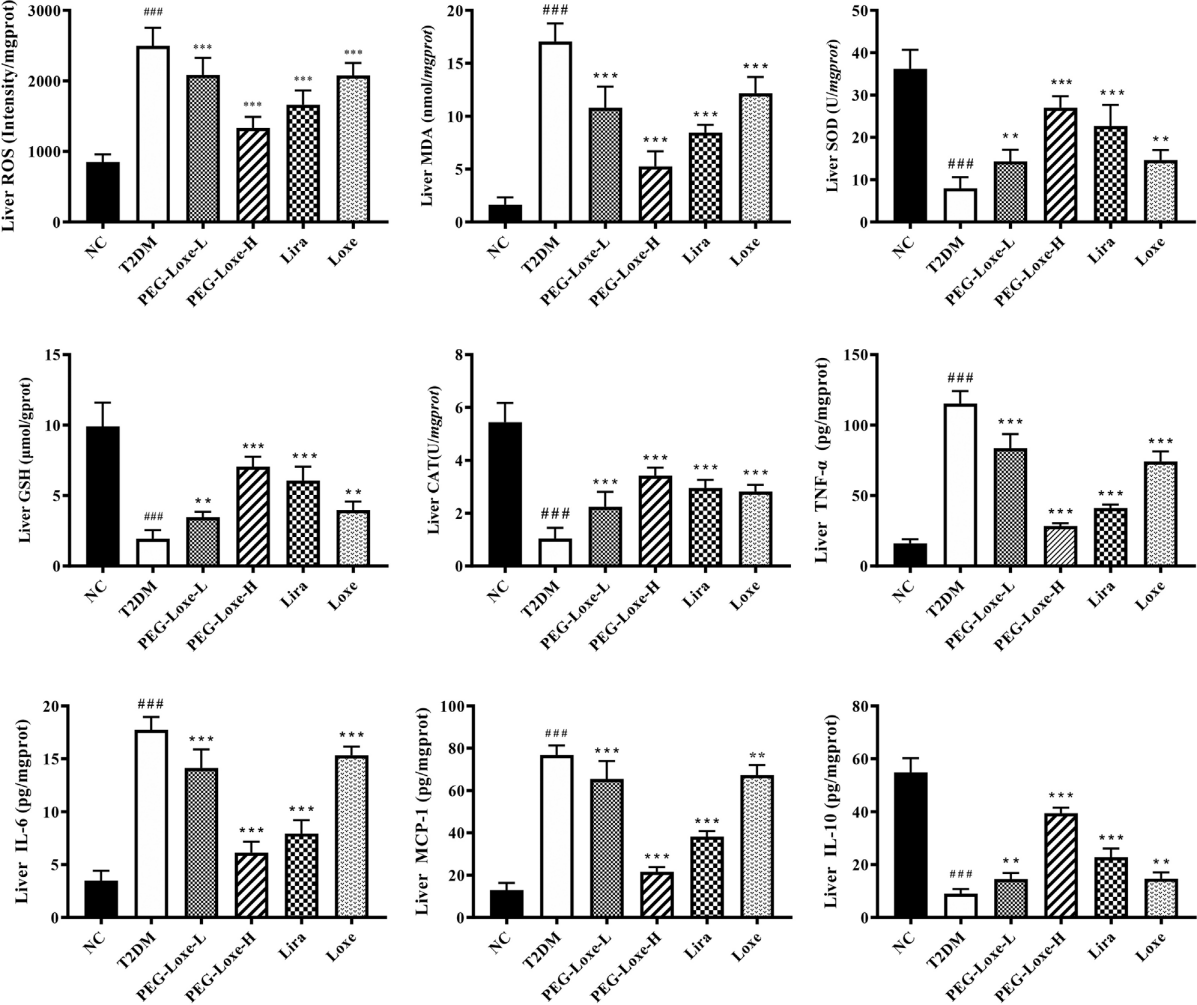
Polyethylene glycol loxenatide (PEG-Loxe) improved oxidative stress and the production of inflammatory factors in the liver of *db/db* mice. Oxidative stress parameters, including reactive oxygen species (ROS), malondialdehyde (MDA), glutathione (GSH), superoxide dismutase (SOD), and catalase (CAT), and inflammatory factors, including tumor necrosis factor-α (TNF-α), interleukin-6 (IL-6), monocyte chemotactic protein-1 (MCP-1), and interleukin-10 (IL-10), were detected using the ELISA assay. Data are presented as the mean ± SD; n = 8; ^###^ *p* < 0.001 compared to the normal control group; ^**^ *p* < 0.01 and ^***^ *p* < 0.001 compared to the T2DM group.

**FIGURE 4:**
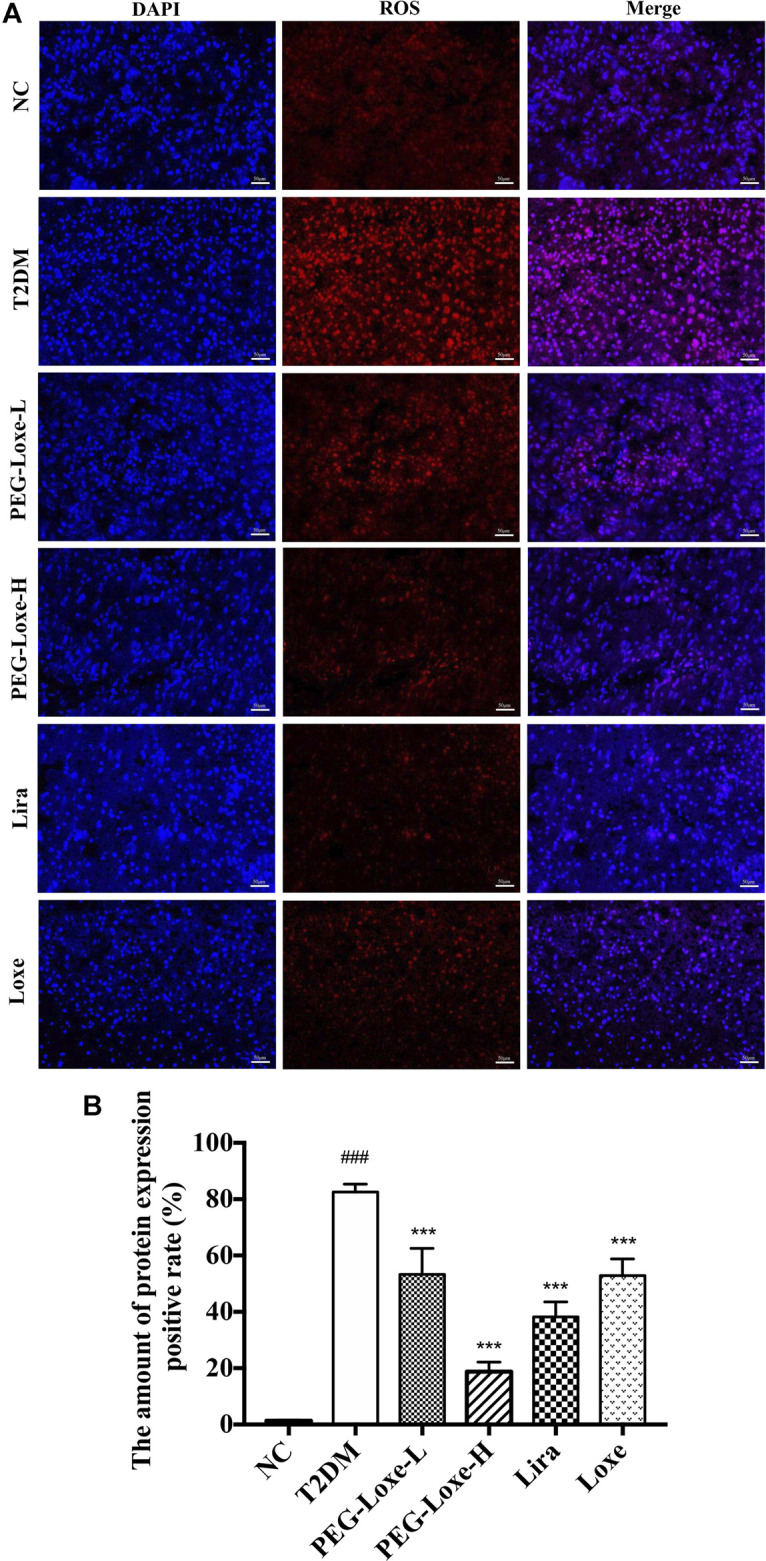
Polyethylene glycol loxenatide (PEG-Loxe) reduced ROS levels in *db/db* mice (A) Representative images of reactive oxygen species (ROS) levels after dihydroethidium (DHE) staining (200×) (B) ROS levels expressed as the ratio of the fluorescence intensity of the DHE-positive area to 4′,6′-diamidino-2-phenylindole (DAPI). Data are presented as the mean ± SD; n = 3; ^##^ *p* < 0.01 and ^###^ *p* < 0.001 compared to the normal control group; ^*^ *p* < 0.05, ^**^ *p* < 0.01, and ^***^ *p* < 0.001 compared to the T2DM group.

Our further exploration of the potential mechanism of PEG-Loxe’s protection against liver damage revealed that T2DM mice had significantly higher liver ROS and MDA levels and pro-inflammatory factors TNF-α, IL-6, and MCP-1 levels than normal mice and markedly lower levels of liver antioxidant enzymes SOD, GSH, and CAT and anti-inflammatory cytokine IL-10 (Figure 3), indicating that oxidative stress and inflammation possibly occur with long-term hyperglycemic stimulation of the liver. Figure 4 shows significantly enhanced ROS fluorescence intensity and drastically elevated ROS content in the liver tissues of T2DM mice, consistent with the results of our biochemical experiments. In contrast, PEG-Loxe, Lira, and Loxe reversed the above changes, indicating that PEG-Loxe protected the liver by reducing hepatic oxidative stress and inflammatory damage in T2DM mice.

### PEG-Loxe Regulates Lipid Metabolism Through the Sirt1-AMPK Pathway to Improve Liver Damage in T2DM Mice

**FIGURE 5:**
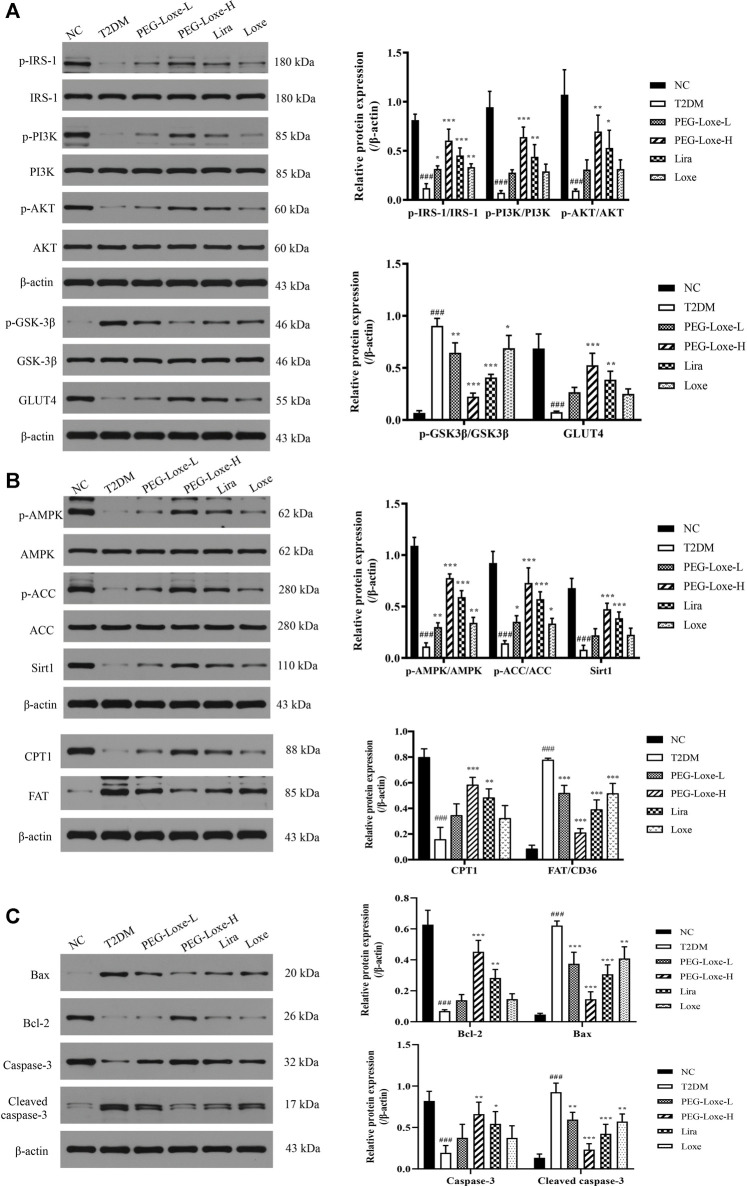
Polyethylene glycol loxenatide (PEG-Loxe) regulates the expressions of the Sirt1-AMPK pathway-, insulin PI3K/AKT pathway- and apoptosis-related proteins. Protein expression levels were normalized to the levels of β-actin. Data are presented as the mean ± SD; n = 3; ^###^ *p* < 0.001 compared to the normal control group; ^*^ *p* < 0.05, ^**^ *p* < 0.01, and ^***^ *p* < 0.001 compared to the T2DM group.

The Sirt1-AMPK signaling pathway is crucial to glucolipid metabolism in the body, but it also causes oxidative stress and inflammation (Jung et al., 2015). We assessed the effect of PEG-Loxe on the hepatic Sirt1-AMPK pathway in T2DM mice. As shown in Figure 5B, hepatic Sirt1, p-AMPK, p-ACC and CPT1 expressions decreased and FAT increased significantly in T2DM mice compared to normal mice (*p* < 0.001). However, treatment with PEG-Loxe and Lira improved markedly, suggesting that PEG-Loxe can inhibit hepatic lipid synthesis and oxidative stress, and promote fatty acid oxidation by regulating lipid metabolism via the Sirt1-AMPK pathway, thereby improving liver damage in T2DM.

### PEG-Loxe Improves Pancreatic Islet Damage, Increases Pancreatic GLP-1R Expression, and Promotes Pancreatic Insulin Secretion in T2DM Mice

**FIGURE 6:**
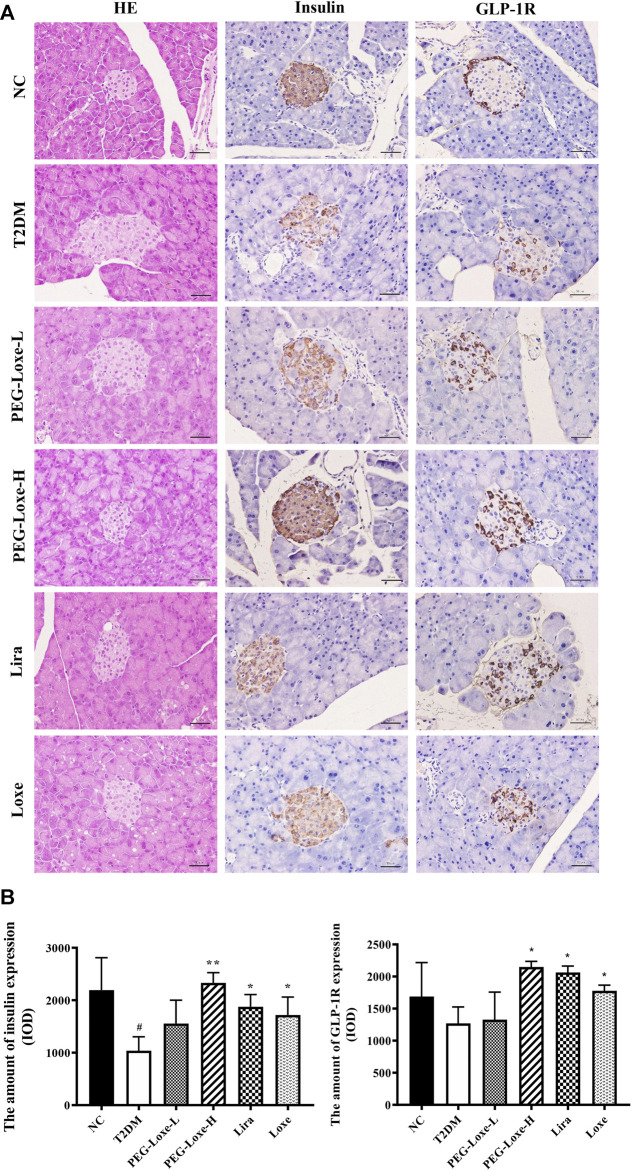
Polyethylene glycol loxenatide (PEG-Loxe) prevents injury to pancreatic islets and promotes insulin secretion and GLP-1R production in *db/db* mice **(A)** Histological examination of pancreatic islet slices with hematoxylin and eosin (H&E) staining (400×) and representative images of islet insulin and glucagon-like peptide-1 receptor (GLP-1R) staining by the immunohistochemistry test (400×) **(B)** Quantitative immunohistochemical analysis of insulin and GLP-1R levels in islets. Data are presented as the mean ± SD; n = 3; ^#^ *p* < 0.05 compared to the normal control group; ^*^ *p* < 0.05 and ^**^ *p* < 0.01 compared to the T2DM group.

As shown in Figure 6A, the H&E staining revealed that the islets of the pancreatic tissues of mice in the NC group had regular morphology (round and oval) with clear borders and uniform distribution of islet cells (at ×400 magnification). On the other hand, the islets of mice in the T2DM group were swollen, irregular in morphology (polygonal), and had blurred borders. The morphologies of the islets of mice in the PEG-Loxe, Lira, and Loxe treatment groups showed improvement compared to those of T2DM group mice. PEG-Loxe is an agonist of GLP-1R, capable of promoting the synthesis and release of insulin by binding to GLP-1R. As shown in Figures 6A,B, PEG-Loxe-H, Lira, and Loxe significantly increased pancreatic GLP-1R levels in T2DM mice compared to T2DM mice, thereby promoting insulin secretion. Consistently, the islet insulin levels in the T2DM group were significantly lower than those in the NC group, indicating that the islet β-cells of T2DM mice had a reduced ability to secrete insulin and an impaired islet function. PEG-Loxe-H, Lira, and Loxe markedly increased insulin levels in T2DM mice (Figure 6B, *p* < 0.05). These results suggest that PEG-Loxe-H, Lira, and Loxe can repair damaged islet cells and promote insulin secretion from pancreatic β-cells, thus exerting hypoglycemic effects.

### PEG-Loxe Inhibits Pancreatic β-Cell Apoptosis in T2DM Mice

To understand whether the damage of pancreatic islets was caused by β-cell apoptosis, we determined the expression of apoptosis-related proteins in pancreatic tissues using western blot. Our findings showed that the anti-apoptotic protein, Bcl-2, was significantly lower and the pro-apoptotic proteins, Bax and Cleaved-caspase-3, were considerably higher in the T2DM group of mice than in the NC group, pointing to increased apoptosis of islet cells in T2DM mice. PEG-Loxe, Lira, and Loxe drastically decreased Bax and Cleaved-caspase-3 levels while stimulating Bcl-2 expression, effectively inhibiting β-cell apoptosis (Figure 5C).

### PEG-Loxe Activates the Insulin Signaling Pathway and Increases Serum Insulin Levels in T2DM Mice

As shown in Table 1, serum insulin levels were significantly higher in T2DM mice than in normal mice (about 2-fold higher), and treatment with PEG-Loxe-L, PEG-Loxe-H, and Lira further significantly increased serum insulin levels by 12.94, 26.80, 12.24%, respectively (*p* < 0.05).

To further investigate the impact of PEG-Loxe on insulin signaling, the expressions of proteins associated with the hepatic PI3K/AKT pathway were determined using western blot. The hepatic insulin signaling pathway is initiated when insulin binds to insulin receptor-β and is then activated by insulin receptor substrate (IRS)-1, followed by the triggering of the PI3K/AKT pathway, prompting the transfer of glucose transporter protein (GLUT) from the cytoplasm to the cell membrane to promote glucose absorption and glycogen synthesis (Wang et al., 2018). Proteins p-IRS-1, p-PI3K, p-AKT, and GLUT4 expression in the liver were significantly lower while p-GSK-3β expression was considerably higher in T2DM mice compared to the NC group, indicating that the insulin pathway in T2DM mice was impeded. PEG-Loxe, Lira, and Loxe activated the hepatic insulin signaling pathway in T2DM mice, as evidenced by pointedly elevated p-IRS-1, p-PI3K, p-AKT, and GLUT4 levels and considerably diminished p-GSK-3β expression (Figure 5A).

**FIGURE 7:**
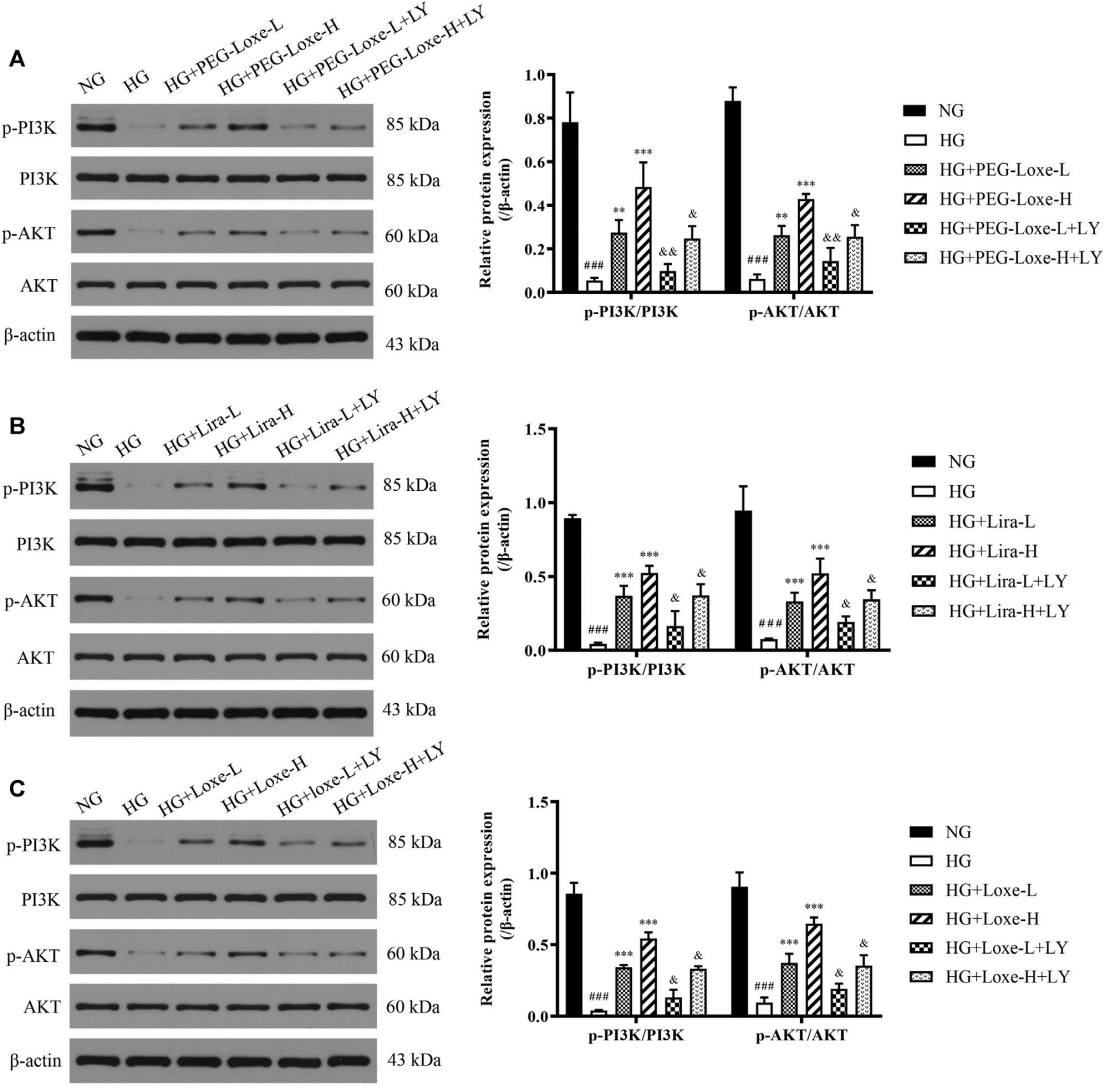
The expressions of phospho-phosphoinositol 3 kinase (p-PI3K), PI3K, phospho-protein kinase B (p-AKT), and AKT in HepG2 cells treated with normal glucose (NG), high glucose (HG), HG + PEG-Loxe, HG + PEG-Loxe + LY (PI3K inhibitor), HG + Lira, HG + Lira + LY, HG + Loxe, and HG + Loxe + LY. Protein expression levels were normalized to the levels of β-actin. Data are presented as the mean ± SD; n = 3; ^###^ *p* < 0.001 compared to the NG group; ^**^ *p* < 0.01 and ^***^ *p* < 0.001 compared to the HG group; ^&^ *p* < 0.05 and ^&&^ *p* < 0.01 compared to the HG + PEG-Loxe/HG + Lira/HG + Loxe group.

*In vitro* experiments were performed to further verify whether PEG-Loxe activated the PI3K/AKT signaling pathway. Results were shown in Figures 7A–C p-PI3K/PI3K and p-AKT/AKT in HepG2 cells were significantly lowered after high glucose culture (*p* < 0.001), indicating that the PI3K/AKT signaling pathway was impeded. HepG2 cells in the PEG-Loxe, Lira, and Loxe groups had significantly higher levels of each protein compared to the high glucose group (*p* < 0.01). In the presence of the PI3K inhibitor LY294002, p-PI3K/PI3K and p-AKT/AKT protein levels decreased substantially (*p* < 0.05). The addition of LY294002 offset the activation of the insulin pathway by PEG-Loxe, Lira, and Loxe, further confirming that PEG-Loxe can activate the PI3K/AKT insulin signaling pathway.

## Discussion

GLP-1RAs are novel hypoglycemic agents that have emerged in recent years and are now the focus of clinical studies for the treatment of T2DM because they have specific outstanding advantages (Müller et al., 2019). Our results suggest that PEG-Loxe can effectively lower blood glucose and improve glucolipid disorders in *db/db* mice. However, the underlying mechanisms had not been explored. Therefore, in the present study, we sought to establish the mechanism of the hypoglycemic and hypolipidemic effects of PEG-Loxe using T2DM as a model.

The liver is the central organ for glycogen synthesis and glucose metabolism. Insulin resistance, hyperglycemia, and disorders of fatty acid metabolism are important causes of dyslipidemia after meals in diabetic patients. In 2020, an international panel of 30 experts from 22 countries published a consensus that the diagnostic criteria for metabolic dysfunction-associated fatty liver disease (MAFLD) is based on hepatic fat accumulation in combination with one of the following three conditions: overweight/obesity, type 2 diabetes, or metabolic dysfunction. Among them, triacylglycerol (TAG) accumulation was a risk factor for metabolic abnormalities (Eslam et al., 2020). Studies have shown that liraglutide, exenatide, and lixisenatide reduce TG, TC, and LDL-C in the blood of diabetic patients during T2DM treatment (Voukali et al., 2014; Sun et al., 2015; Roca-Rodríguez et al., 2017). In the meanwhile, these drugs also protect against T2DM-induced hepatic steatosis and liver damage by inhibiting oxidative stress and various inflammatory responses, and promoting body mass reduction in patients. Consistent with previous reports, we also observed a significant reduction in hepatic TC, TG and serum TG levels in *db/db* mice after 4 weeks of treating mice with PEG-Loxe, Lira, and Loxe in the present study. Furthermore, we also assessed and observed a significant increase in liver AST and ALT levels in T2DM mice, pointing to liver tissue damage. Histopathological evaluations also revealed steatosis, cytoplasmic vacuolation, massive lipid droplet deposition, and slight fibrosis in liver tissue sections, further confirming the accumulation of fat in the liver and the impaired structure and function of the liver. However, treatment with PEG-Loxe, Lira, and Loxe reversed these pathological changes. PEG-Loxe-L exhibited comparable effects to those of Lira in lowering blood glucose and regulating lipid levels, suggesting that PEG-Loxe could also improve diabetes-induced hepatic lipid disorders and liver damage, which is corresponding to previous literature reports (Trevaskis et al., 2012; Liu et al., 2015).

Oxidative stress and inflammatory response are critical for abnormal glucolipid metabolism in T2DM and are also the main reason behind the progression of diabetes and its complications. Chronic high-sugar and high-fat diets lead to excessive hepatic lipid deposition, oxidative stress, and inflammatory response (Zhou et al., 2019). In this study, we observed similar changes in the levels of oxidative and inflammatory factors in diabetic mice, with ROS fluorescence intensity in liver tissues significantly enhanced, suggesting that chronic hyperglycemia stimulates peroxidative and inflammatory damages in the liver. Treatment with PEG-Loxe, Lira, and Loxe notably repaired these abnormal changes, as shown by the decline in ROS, MDA, TNF-α, IL-6, and MCP-1 levels and the increase in SOD, GSH, CAT, and IL-10 levels. Of these treatments, PEG-Loxe modulations on the above factors were dose-dependent, and PEG-Loxe-L and Loxe had comparable weaker remediation impact on hepatic oxidative stress and inflammatory response than Lira. In line with these findings, one investigation established that GLP-1 effectively improved endothelial dysfunction and enhanced antioxidant and anti-inflammatory levels in T2DM patients (Ceriello et al., 2014). Overall, our results suggest that PEG-Loxe possibly reduces lipid metabolism disorders in T2DM and protects against diabetes-induced liver damage by activating antioxidant defense systems and attenuating inflammatory responses.

Lipid metabolism disorder can cause the deposition of TAG and fatty acids in hepatocytes, and then promote the occurrence and development of fatty liver. In clinical practice, GLP-1RAs reduce body weight and liver fat accumulation. However, there is limited research on the underlying mechanisms. AMPK is an important signaling molecule involved in glucolipid metabolism and is widely distributed in tissues with excited metabolisms, such as the liver, adipose tissues, and skeletal muscles. ACC is a downstream target of AMPK regulation, with the AMPK/ACC pathway playing a key role as a regulator of energy homeostasis, including mitochondrial biogenesis, cellular lipolysis, fatty acid synthesis and oxidation (Hardie et al., 2012). In the liver, GLP-1 stimulated the phosphorylation of AMPK and expression of Sirt1, then activated AMPK can phosphorylate ACC and reduce the production of malonyl-coA, thus up-regulating CPT1 expression, reducing FAT level, promoting fatty acid oxidation and reducing lipid deposition (Mottillo et al., 2016; Liu et al., 2015; Wan et al., 2021; O'Neill et al., 2013; Ben-Shlomo et al., 2011). Lira, as demonstrated in recent years, improves insulin resistance and hepatic lipid deposition in T2DM rats fed with a high-fat diet and promotes lipid degradation in liver and adipose tissues through activation of the AMPK pathway (He et al., 2016; He et al., 2020; Zhou et al., 2020). Similarly, our study demonstrated that PEG-Loxe, Lira, and Loxe notably upregulated the levels of Sirt1, p-AMPK, and p-ACC in the liver. Activation of AMPK signaling pathway promoted the oxidation of liver fatty acids, which in turn increased CPT1 protein expression, reduced FAT level, and decreased lipid accumulation, with the most significant effect observed in the PEG-Loxe-H group. A previous study demonstrated that the activation of the Sirt1-AMPK pathway inhibits hepatic oxidative stress and inflammatory responses (Jung et al., 2015). Consistent with that observation, our findings suggest that PEG-Loxe possibly inhibits hepatic lipid synthesis, oxidative stress, and inflammatory response, and promote fatty acid oxidation by regulating lipid metabolism via the Sirt1/AMPK/ACC pathway, thereby remedying T2DM liver damage.

The development of T2DM is due in part to damage to the islet β-cells, which results in failure to secrete sufficient insulin (Eizirik et al., 2020). As observed in this study, T2DM mice had swollen islets with irregular morphology and significantly reduced serum insulin levels. Additionally, immunohistochemical measurements of insulin revealed that islet insulin levels were considerably lower in diabetic mice than in normal mice. Therefore, the islets of T2DM mice are damaged and unable to regulate the rise in blood glucose levels. Treatment with PEG-Loxe and Lira could significantly increase pancreatic GLP-1R expression and increase insulin stores, as demonstrated here. Mounting evidence suggests that the reduced number and increased apoptosis of islet β-cells are the primary causes of the impaired structure and function of islets in T2DM (Meier, 2012; Eizirik et al., 2020). GLP-1 promotes islet β-cell proliferation and inhibits β-cell apoptosis (Drucker, 2018). In line with this, PEG-Loxe and Lira could block the pancreatic apoptotic pathway by regulating the expression of apoptotic proteins. As expected, PEG-Loxe treatment markedly increased the expression of the anti-apoptotic protein Bcl-2 and decreased the level of the pro-apoptotic protein Bax, which led to an increase in the Bcl-2/Bax ratio, resulting in the inhibition of the apoptosis enforcer Cleaved-caspase-3/Caspase-3 ratio and the blockage of the β-cell apoptosis program. PEG-Loxe tellingly inhibits β-cell apoptosis arguably by restoring the Bcl-2/Bax balance, blocking the endogenous apoptotic pathway, promoting the recovery of islet structural breakdowns and functional defects, and increasing insulin secretion for the effective treatment of T2DM.

The impairment of the PI3K/AKT signaling pathway is the key to causing glucose metabolism disorders. GLP-1R, which belongs to the G protein-coupled receptor family, exerts biological effects mainly through the activation of phosphoinositide 3 kinase (PI3K), protein kinase A (PKA), and extracellular signal-regulated kinase (ERK) 1/2 (He et al., 2019; Li et al., 2019). GLP-1RAs activate the PI3K/AKT and AMPK signaling pathways in T2DM rats, protecting against T2DM-related learning memory impairment and lowering blood glucose (Yang et al., 2018). Here, T2DM mice harbored an impaired hepatic insulin pathway and had reduced serum insulin levels, which led to an increase in blood glucose. However, PEG-Loxe, Lira, and Loxe significantly increased serum insulin levels, and the expression of hepatic proteins p-IRS-1, p-PI3K, p-AKT, and GLUT4 in T2DM mice. *In vitro* cellular experiments confirmed that the activation of the insulin pathway by PEG-Loxe, Lira, and Loxe was offset by the presence of the PI3K inhibitor, further demonstrating that PEG-Loxe, Lira, and Loxe promote insulin secretion and stimulate the hepatic insulin pathway, thereby improving insulin sensitivity and promoting glucose uptake, which eventually reduces blood glucose.

## Conclusion

In summary, we have shown that PEG-Loxe has hypoglycemic and hepatoprotective effects in db/db mice. PEG-Loxe inhibited hepatic lipid synthesis, oxidative stress, and inflammatory response by activating lipid metabolism through the Sirt1/AMPK/ACC pathway, thereby improving T2DM-associated liver damage. PEG-Loxe also subdued β-cell apoptosis, increased pancreatic GLP-1R expression, and activated the insulin PI3K/AKT pathway, promoting the synthesis and secretion of insulin and providing hypoglycemic effects.

## Data Availability

The original contributions presented in the study are included in the article/supplementary material, further inquiries can be directed to the corresponding authors.
